# The genome sequence of the marsh cinquefoil, *Comarum palustre* L., also known as *Potentilla palustris* (L.) Scop. (Rosaceae)

**DOI:** 10.12688/wellcomeopenres.23016.1

**Published:** 2024-09-05

**Authors:** Maarten J. M. Christenhusz, Ilia J. Leitch, Royal Botanic Gardens Kew Genome Acquisition Lab, Darwin Tree of Life Barcoding collective, Plant Genome Sizing collective, Wellcome Sanger Institute Tree of Life Management, Samples and Laboratory team, Wellcome Sanger Institute Scientific Operations: Sequencing Operations, Wellcome Sanger Institute Tree of Life Core Informatics team, Tree of Life Core Informatics collective, Darwin Tree of Life Consortium

**Affiliations:** 1 Royal Botanic Gardens Kew, Richmond, England, UK; 2 Curtin University, Perth, Western Australia, Australia

**Keywords:** Comarum palustre, marsh cinquefoil, genome sequence, chromosomal, Rosales

## Abstract

We present a genome assembly from an individual *Comarum palustre* (the marsh cinquefoil; Streptophyta; Magnoliopsida; Rosales; Rosaceae). The genome sequence has a total length of 528.90 megabases. Most of the assembly is scaffolded into 21 chromosomal pseudomolecules suggesting the individual is an allohexaploid (2 *n* = 6 *x* = 42). The mitochondrial and plastid genome assemblies have lengths of 362.32 kilobases and 154.29 kilobases, respectively. Gene annotation of this assembly on Ensembl identified 37,459 protein-coding genes.

## Species taxonomy

Eukaryota; Viridiplantae; Streptophyta; Streptophytina; Embryophyta; Tracheophyta; Euphyllophyta; Spermatophyta; Magnoliopsida; Mesangiospermae; eudicotyledons; Gunneridae; Pentapetalae; rosids; fabids; Rosales; Rosaceae; Rosoideae; Potentilleae; Fragariinae; *Comarum*; *Comarum palustre* L. (NCBI:txid57932).

## Background

**Figure 1.**
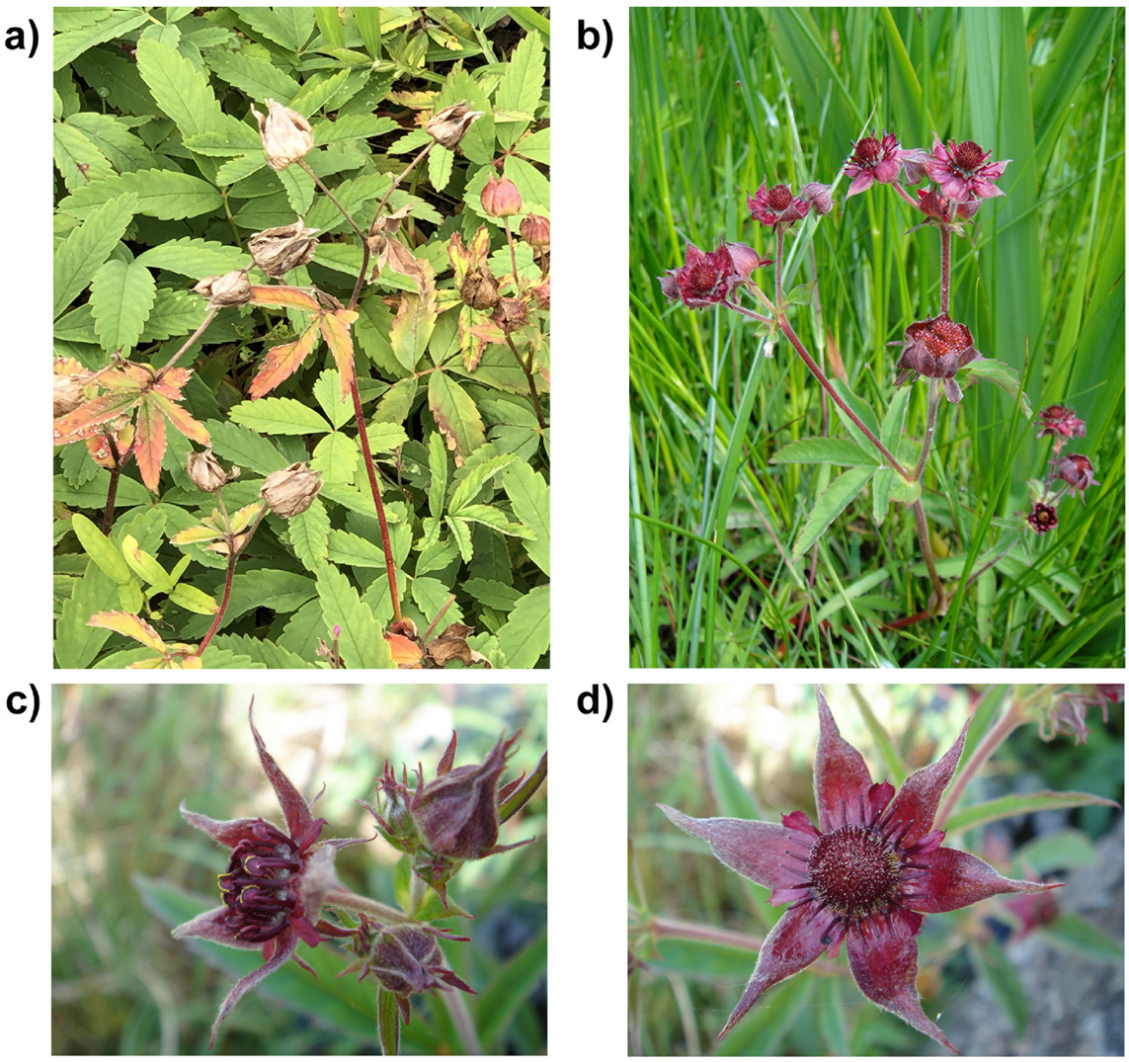
Photographs of *Comarum palustre*. **a**). The specimen used for genome sequencing (drComPalu1), ( **b**) the plant growing in the wild, ( **c** and **d**) two close ups of the flower.

*Comarum palustre* (Rosaceae), also known as *Potentilla palustris*, is a widespread perennial aquatic herb, found across the subarctic and temperate Northern Hemisphere ( POWO, 2024) in bogs, along margins of streams and ponds and in wet meadows. It has creeping, ascending stems, often floating on the water surface, with alternate three- to seven-parted leaves and flowers with wine-red sepals and petals ( Figure 1 b to d)). It is common in Ireland, Wales, Scotland and northern England, but there less so in the south. It prefers peaty or sandy soils and neutral to acidic water to thrive. Marsh cinquefoil is regularly cultivated as a pond margin ornamental, and in herbalism it has been used to promote digestion, although little scientific research has confirmed it to be effective.

As previously discussed in the genome note on silverweed ( *Potentilla anserina* L., Christenhusz *et al*., 2023), the taxonomy of the genus *Potentilla* L. is contentious. Based on molecular phylogenetic studies using nuclear and plastid markers ( Eriksson *et al.*, 1998; Eriksson *et al.*, 2022; Persson, 2021; Töpel *et al.*, 2011), it is evident that the genus in the traditional sense is polyphyletic, with diverse genera such as *Alchemilla* L., *Fragaria* L. and *Comarum* L. embedded in it. Therefore, the choice between recognising this as a species of *Potentilla* or separating it in its own genus *Comarum* is a matter of how widely or narrowly *Potentilla* should be circumscribed. Currently, the NCBI taxonomy database recognises this species as belonging to *Comarum* ( Schoch *et al.*, 2020). If *Comarum* is recognised as a distinct genus within the subtribe Fragariineae, it also necessitates accepting *Anserina* and *Dasiphora*, and a few others as genera, whereas inclusion of these within *Potentilla* should result in the inclusion of other well-known genera such as *Fragaria* (strawberries) *and Alchemilla* (lady’s mantles). Here we provide both names until the nomenclature of this group is more fully resolved. Nevertheless, it is noted that *Comarum* is likely to be closely related to *Fragaria* as hybrids between *Comarum* and *Fragaria* ( *× Comagaria* Büscher & G.H.Loos) are frequent in horticulture. Indeed, this further calls into question the validity of the generic circumscription ( Ellis, 1962; Mabberley, 2002).

*Comarum palustre* is known to have a variety of ploidy levels built on a base number of *x* = 7 chromosomes. The vast majority of individuals studied across its range are reported to be hexaploid with 2 *n* = 6 *x =* 42 (e.g. Knaben & Engelskjøn, 1967; Lövkvist & Hultgård, 1999), although, to date, hexaploids have not been recorded in Britain or Ireland, where instead the few counts revealed either pentaploid (2 *n* = 5 *x* = 35) or nonaploid (2 *n* = ~9x = 64) individuals ( Dempsey *et al.*, 1994). Other ploidy levels recorded include tetraploid specimens (2 *n* = 4x = 28) from Iceland, Central and Northern Europe and Siberia ( Ehrenberg, 1945; Löve & Löve, 1956; Wulff, 1937) and an octoploid specimen (2 *n* = 8 *x* = 56) found in Norway ( Laane & Lie, 1985).

Here we present a chromosomal-level whole genome sequence for *Comarum palustre*, based on a specimen grown at the Royal Botanic Gardens, Kew. This high-quality genome will be excellent for helping to understand the evolutionary relationships between this species and other members of the variable tribe Potentillinae. It could potentially also be used to find genes that help in pest resistance for strawberry breeding.

## Genome sequence report

The genome of *Comarum palustre* ( Figure 1) was sequenced using Pacific Biosciences single-molecule HiFi long reads, generating a total of 22.42 Gb (gigabases) from 2.66 million reads, providing approximately 35-fold coverage. Using flow cytometry, the genome size (1C-value) was estimated to be 0.70 pg, equivalent to 680 Mb. Primary assembly contigs were scaffolded with chromosome conformation Hi-C data, which produced 187.37 Gb from 1,240.85 million reads, yielding an approximate coverage of 354-fold. Specimen and sequencing information is summarised in Table 1.

**Table 1.** Specimen and sequencing data for *Comarum palustre*.

Project information			
**Study title**	Comarum palustre		
**Umbrella BioProject**	PRJEB61204		
**Species**	*Comarum palustre*		
**BioSample**	SAMEA7522399		
**NCBI taxonomy ID**	57932		
Specimen information			
**Technology**	**ToLID**	**BioSample accession**	**Organism part**
**PacBio long read sequencing**	drComPalu1	SAMEA7522439	leaf
**Hi-C sequencing**	drComPalu1	SAMEA7522441	leaf
**RNA sequencing**	drComPalu1	SAMEA7522439	leaf
Sequencing information			
**Platform**	**Run accession**	**Read count**	**Base count (Gb)**
**Hi-C Illumina NovaSeq 6000**	ERR11217133	1.24e+09	187.37
**PacBio Sequel IIe**	ERR11242118	2.66e+06	22.42
**RNA Illumina NovaSeq 6000**	ERR12321225	6.17e+07	9.32

**Figure 2.**
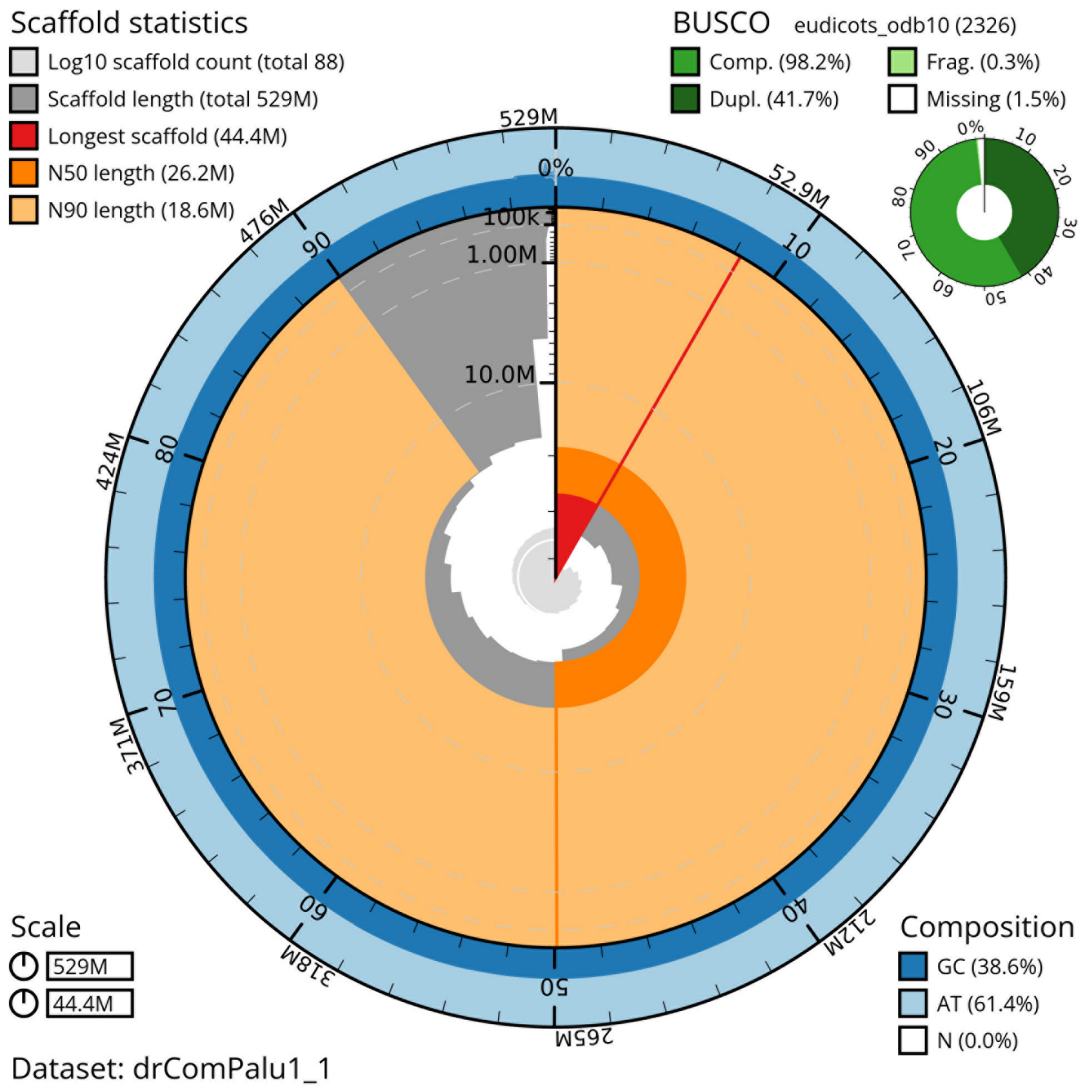
Genome assembly of *Comarum palustre*, drComPalu1.1: metrics. The BlobToolKit snail plot shows N50 metrics and BUSCO gene completeness. The main plot is divided into 1,000 size-ordered bins around the circumference with each bin representing 0.1% of the 529,403,557 bp assembly. The distribution of scaffold lengths is shown in dark grey with the plot radius scaled to the longest scaffold present in the assembly (44,384,015 bp, shown in red). Orange and pale-orange arcs show the N50 and N90 scaffold lengths (26,215,688 and 18,625,543 bp), respectively. The pale grey spiral shows the cumulative scaffold count on a log scale with white scale lines showing successive orders of magnitude. The blue and pale-blue area around the outside of the plot shows the distribution of GC, AT and N percentages in the same bins as the inner plot. A summary of complete, fragmented, duplicated and missing BUSCO genes in the eudicots_odb10 set is shown in the top right. An interactive version of this figure is available at https://blobtoolkit.genomehubs.org/view/drComPalu1_1/dataset/drComPalu1_1/snail.

**Figure 3.**
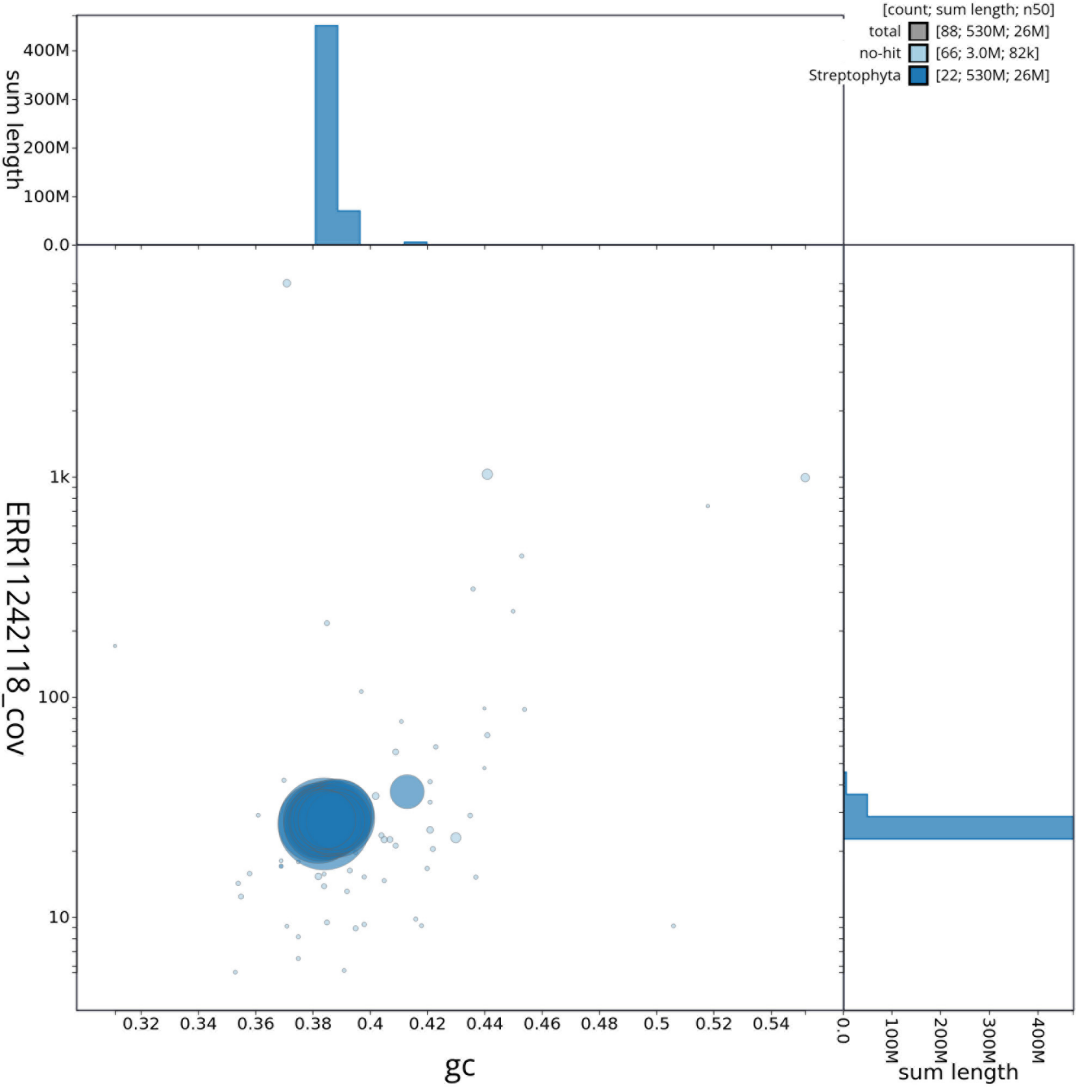
Genome assembly of *Comarum palustre*, drComPalu1.1: Blob plot of base coverage in ERR11242118 against GC proportion for sequences in the assembly. Sequences are coloured by phylum. Circles are sized in proportion to sequence length. Histograms show the distribution of sequence length sum along each axis. An interactive version of this figure is available at https://blobtoolkit.genomehubs.org/view/drComPalu1_1/dataset/drComPalu1_1/blob.

**Figure 4.**
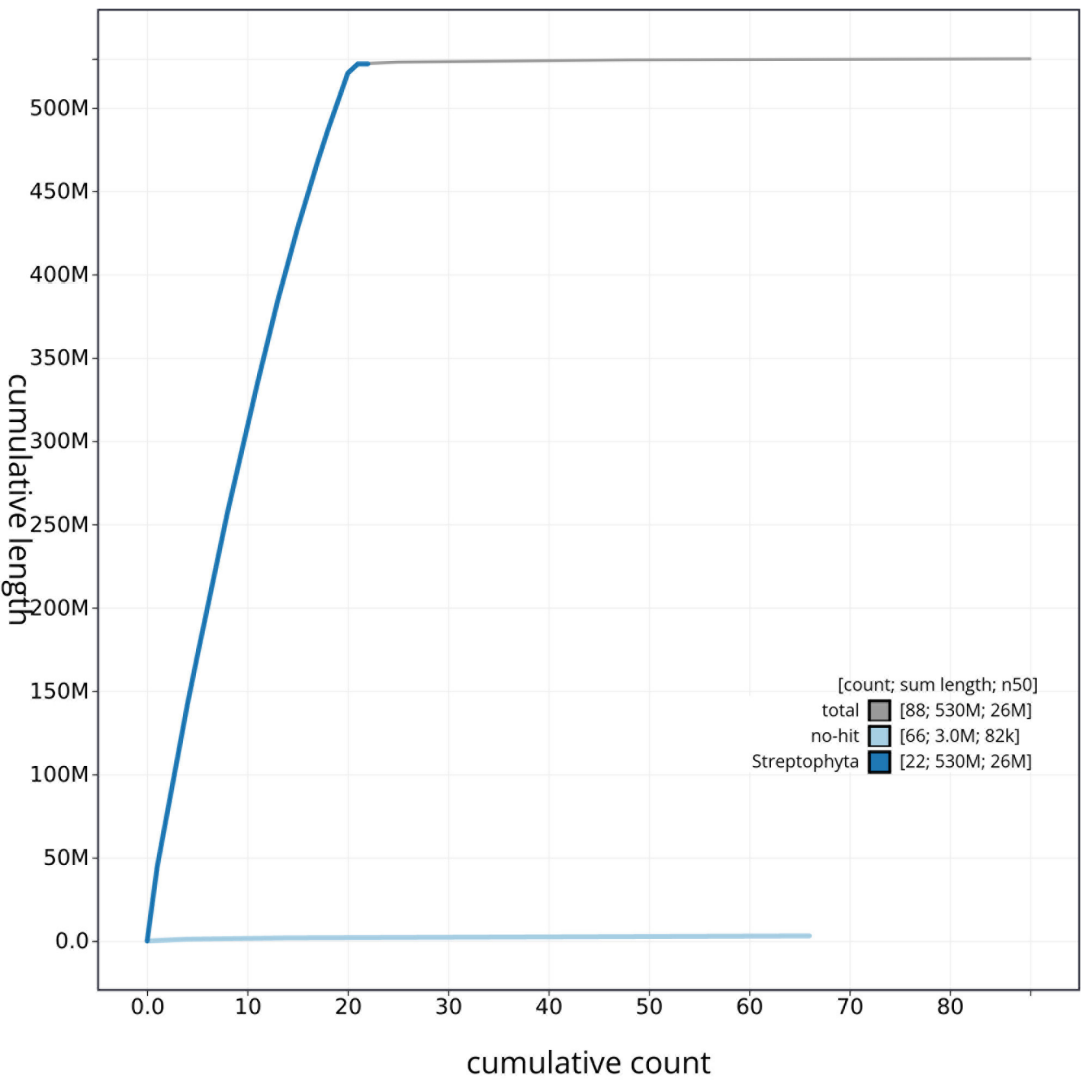
Genome assembly of *Comarum palustre* drComPalu1.1: BlobToolKit cumulative sequence plot. The grey line shows cumulative length for all sequences. Coloured lines show cumulative lengths of sequences assigned to each phylum using the buscogenes taxrule. An interactive version of this figure is available at https://blobtoolkit.genomehubs.org/view/drComPalu1_1/dataset/drComPalu1_1/cumulative.

**Figure 5.**
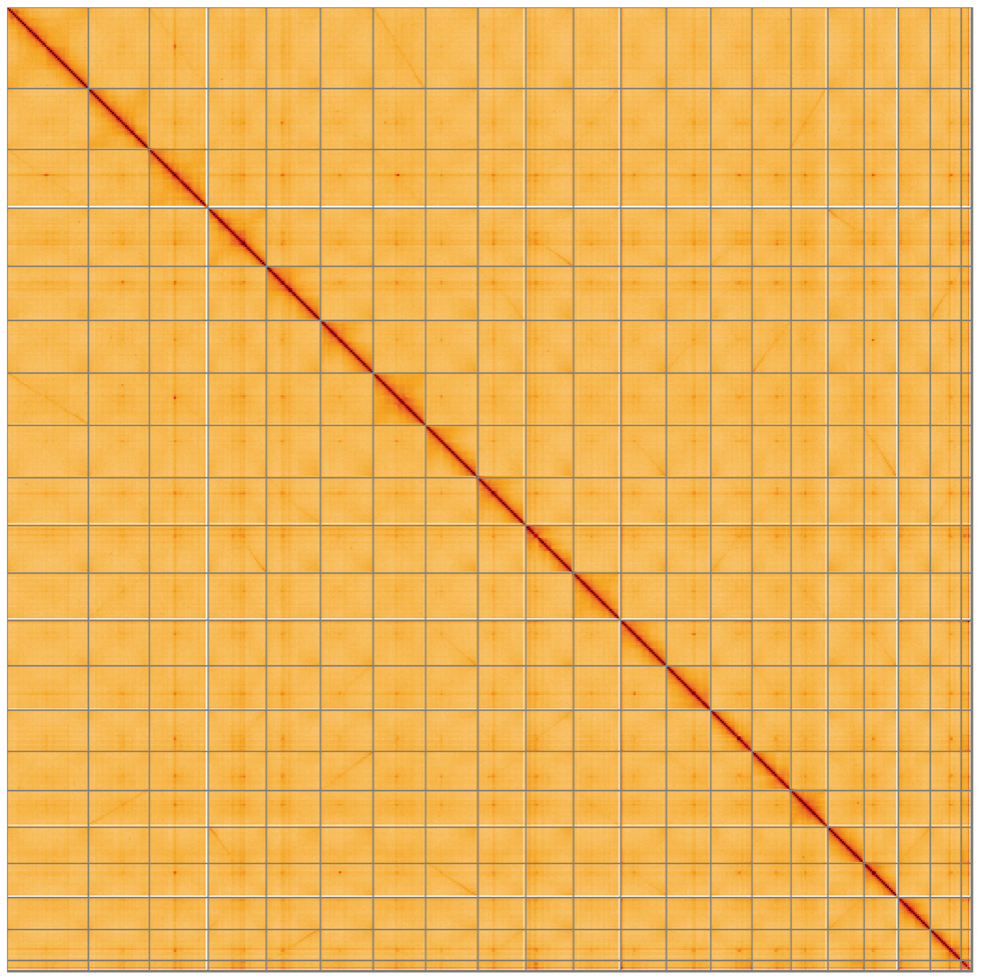
Genome assembly of *Comarum palustre*, drComPalu1.1: Hi-C contact map of the drComPalu1.1 assembly, visualised using HiGlass. Chromosomes are shown in order of size from left to right and top to bottom. An interactive version of this figure may be viewed at https://genome-note-higlass.tol.sanger.ac.uk/l/?d=EN9J7gwbQHSQU9WGMfMU1w.

Manual assembly curation corrected 36 missing joins or mis-joins and 10 haplotypic duplications, reducing the assembly length by 0.33%, and decreasing the scaffold N50 by 8.86%. The final assembly has a total length of 528.90 Mb in 86 sequence scaffolds with a scaffold N50 of 26.2 Mb ( Table 2) with 494 gaps. The snail plot in Figure 2 provides a summary of the assembly statistics, while the distribution of assembly scaffolds on GC proportion and coverage is shown in Figure 3. The cumulative assembly plot in Figure 4 shows curves for subsets of scaffolds assigned to different phyla. Most (99.42%) of the assembly sequence was assigned to 21 chromosomal-level scaffolds. Chromosome-scale scaffolds confirmed by the Hi-C data are named in order of size ( Figure 5; Table 3). Given that the genome sequence assembles into 21 unique chromosome-level scaffolds, the data suggest the individual sequenced is an allohexaploid (2 *n* = 6 *x* = 42). The mitochondrial and plastid genomes were also assembled and can be found as contigs within the multifasta file of the genome submission.

**Table 2.** Genome assembly data for *Comarum palustre*, drComPalu1.1.

Genome assembly		
Assembly name	drComPalu1.1	
Assembly accession	GCA_951800085.1	
*Accession of alternate haplotype*	*GCA_951800075.1*	
Span (Mb)	528.90	
Number of contigs	582	
Contig N50 length (Mb)	1.7	
Number of scaffolds	86	
Scaffold N50 length (Mb)	26.2	
Longest scaffold (Mb)	44.38	
Assembly metrics *		*Benchmark*
Consensus quality (QV)	58.2	*≥ 50*
*k*-mer completeness	99.99%	*≥ 95%*
BUSCO **	C:98.2%[S:56.6%,D:41.7%],F:0.3%,M:1.5%,n:2,326	*C ≥ 95%*
Percentage of assembly mapped to chromosomes	99.42%	*≥ 95%*
Sex chromosomes	None	*localised homologous pairs*
Organelles	Mitochondrial genome: 362.32 kb; plastid genome: 154.29 kb	*complete single alleles*
Genome annotation at Ensembl		
Number of protein-coding genes	37,459	
Number of non-coding genes	6,092	
Number of gene transcripts	57,291	

* Assembly metric benchmarks are adapted from column VGP-2020 of “Table 1: Proposed standards and metrics for defining genome assembly quality” from Rhie *et al.* (2021). ** BUSCO scores based on the eudicots_odb10 BUSCO set using version 5.4.3. C = complete [S = single copy, D = duplicated], F = fragmented, M = missing, n = number of orthologues in comparison. A full set of BUSCO scores is available at https://blobtoolkit.genomehubs.org/view/drComPalu1_1/dataset/drComPalu1_1/busco.

**Table 3.** Chromosomal pseudomolecules in the genome assembly of *Comarum palustre*, drComPalu1.

INSDC accession	Name	Length (Mb)	GC%
OX637572.1	1	44.38	38.5
OX637573.1	2	33.36	38.0
OX637574.1	3	32.0	38.5
OX637575.1	4	31.79	39.0
OX637576.1	5	29.59	38.5
OX637577.1	6	28.76	38.5
OX637578.1	7	28.58	38.5
OX637579.1	8	28.54	38.5
OX637580.1	9	26.22	38.5
OX637581.1	10	25.93	39.0
OX637582.1	11	25.74	38.5
OX637583.1	12	24.94	38.5
OX637584.1	13	24.17	38.5
OX637585.1	14	22.6	39.0
OX637586.1	15	21.37	39.0
OX637587.1	16	20.08	38.5
OX637588.1	17	19.79	38.5
OX637589.1	18	18.63	38.5
OX637590.1	19	17.4	39.0
OX637591.1	20	16.98	38.5
OX637592.1	21	5.5	41.5
OX637593.1	MT	0.36	44.0
OX637594.1	Pltd	0.15	37.0

The estimated Quality Value (QV) of the final assembly is 58.2 with *k*-mer completeness of 99.99%, and the assembly has a BUSCO v5.4.3 completeness of 98.2% (single = 56.6%, duplicated = 41.7%), using the eudicots_odb10 reference set ( *n* = 2,326).

Metadata for specimens, BOLD barcode results, spectra estimates, sequencing runs, contaminants and pre-curation assembly statistics are given at https://links.tol.sanger.ac.uk/species/57932.

## Genome annotation report

The *Comarum palustre* genome assembly (GCA_951800085.1) was annotated at the European Bioinformatics Institute (EBI) on Ensembl Rapid Release. The resulting annotation includes 57,291 transcribed mRNAs from 37,459 protein-coding and 6,092 non-coding genes ( Table 2; https://rapid.ensembl.org/Comarum_palustre_GCA_951800085.1/Info/Index). The average transcript length is 2,906.24. There are 1.32 coding transcripts per gene and 5.44 exons per transcript.

## Methods

### Sample acquisition, DNA barcoding and genome size estimation

A specimen of *Comarum palustre* (specimen ID KDTOL10079, ToLID drComPalu1) was hand-picked on 2020-09-03 from the rock garden at the Royal Botanic Gardens Kew, Surrey, UK (latitude 51.48, longitude –0.29). The origin of the plant was Arisaig, Invernesshire, Scotland, UK. The specimen was collected and identified by Maarten J. M. Christenhusz (Royal Botanic Gardens Kew). The herbarium voucher associated with the sequenced plant is K001400678 and is deposited in the herbarium of RBG Kew (K).

The initial species identification was verified by an additional DNA barcoding process according to the framework developed by Twyford *et al*. (2024). Part of the plant specimen was preserved in silica gel desiccant. A DNA extraction from the dried plant was amplified by PCR for standard barcode markers, with the amplicons sequenced and compared to public sequence databases including GenBank and the Barcode of Life Database (BOLD). The barcode sequences for this specimen are openly available on BOLD ( Ratnasingham & Hebert, 2007). Following whole genome sequence generation, DNA barcodes were also used alongside the initial barcoding data for sample tracking through the genome production pipeline at the Wellcome Sanger Institute ( Twyford *et al.*, 2024). The standard operating procedures for the Darwin Tree of Life barcoding have been deposited on protocols.io ( Beasley *et al.*, 2023).

The genome size was estimated by flow cytometry using the fluorochrome propidium iodide and following the ‘one-step’ method as outlined in Pellicer *et al.* (2021). For this species, the General Purpose Buffer (GPB) supplemented with 3% PVP and 0.08% (v/v) beta-mercaptoethanol was used for isolation of nuclei ( Loureiro *et al.*, 2007), and the internal calibration standard was *Petroselinum crispum* ‘Champion Moss Curled’ with an estimated 1C-value of 2,200 Mb ( Obermayer *et al.*, 2002).

### Nucleic acid extraction

The workflow for high molecular weight (HMW) DNA extraction at the WSI Tree of Life Core Laboratory includes a sequence of core procedures: sample preparation; sample homogenisation, DNA extraction, fragmentation, and clean-up. In sample preparation, the drComPalu1 sample was weighed and dissected on dry ice ( Jay *et al.*, 2023). For sample homogenisation, leaf tissue was cryogenically disrupted using the Covaris cryoPREP ^®^ Automated Dry Pulverizer ( Narváez-Gómez *et al.*, 2023). HMW DNA was extracted using the Automated Plant MagAttract v2 protocol ( Todorovic *et al.*, 2023). HMW DNA was sheared into an average fragment size of 12–20 kb in a Megaruptor 3 system ( Bates *et al.*, 2023). Sheared DNA was purified by solid-phase reversible immobilisation, using AMPure PB beads to eliminate shorter fragments and concentrate the DNA ( Strickland *et al.*, 2023). The concentration of the sheared and purified DNA was assessed using a Nanodrop spectrophotometer and Qubit Fluorometer and Qubit dsDNA High Sensitivity Assay kit. Fragment size distribution was evaluated by running the sample on the FemtoPulse system.

RNA was extracted from leaf tissue of drComPalu1 in the Tree of Life Laboratory at the WSI using the RNA Extraction: Automated MagMax™ *mir*Vana protocol ( do Amaral *et al.*, 2023). The RNA concentration was assessed using a Nanodrop spectrophotometer and a Qubit Fluorometer using the Qubit RNA Broad-Range Assay kit. Analysis of the integrity of the RNA was done using the Agilent RNA 6000 Pico Kit and Eukaryotic Total RNA assay.

Protocols developed by the WSI Tree of Life core laboratory are publicly available on protocols.io ( Denton *et al.*, 2023).

### Sequencing

Pacific Biosciences HiFi circular consensus DNA sequencing libraries were constructed according to the manufacturers’ instructions. Poly(A) RNA-Seq libraries were constructed using the NEB Ultra II RNA Library Prep kit. DNA and RNA sequencing was performed by the Scientific Operations core at the WSI on Pacific Biosciences Sequel IIe (HiFi) and Illumina NovaSeq 6000 (RNA-Seq) instruments. Hi-C data were also generated from leaf tissue of drComPalu1 using the Arima-HiC v2 kit. The Hi-C sequencing was performed using paired-end sequencing with a read length of 150 bp on the Illumina NovaSeq 6000 instrument.

### Genome assembly, curation and evaluation

***Assembly***

The original assembly of HiFi reads was performed using Hifiasm ( Cheng *et al.*, 2021) with the --primary option. Haplotypic duplications were identified and removed with purge_dups ( Guan *et al.*, 2020). Hi-C reads were further mapped with bwa-mem2 ( Vasimuddin *et al.*, 2019) to the primary contigs, which were further scaffolded using the provided Hi-C data ( Rao *et al.*, 2014) in YaHS ( Zhou *et al.*, 2023) using the --break option. Scaffolded assemblies were evaluated using Gfastats ( Formenti *et al.*, 2022), BUSCO ( Manni *et al.*, 2021) and MERQURY.FK ( Rhie *et al.*, 2020).

The organelle genomes were assembled using MitoHiFi ( Uliano-Silva *et al.*, 2023) and OATK ( Zhou, 2023).

***Curation***

The assembly was decontaminated using the Assembly Screen for Cobionts and Contaminants (ASCC) pipeline (article in preparation). Manual curation was primarily conducted using PretextView ( Harry, 2022), with additional insights provided by JBrowse2 ( Diesh *et al.*, 2023) and HiGlass ( Kerpedjiev *et al.*, 2018). Scaffolds were visually inspected and corrected as described by Howe *et al*. (2021). Any identified contamination, missed joins, and mis-joins were corrected, and duplicate sequences were tagged and removed. The process is documented at https://gitlab.com/wtsi-grit/rapid-curation (article in preparation).

***Evaluation of final assembly***

A Hi-C map for the final assembly was produced using bwa-mem2 ( Vasimuddin *et al.*, 2019) in the Cooler file format ( Abdennur & Mirny, 2020). To assess the assembly metrics, the *k*-mer completeness and QV consensus quality values were calculated in Merqury ( Rhie *et al.*, 2020). This work was done using the “sanger-tol/readmapping” ( Surana *et al.*, 2023a) and “sanger-tol/genomenote” ( Surana *et al.*, 2023b) pipelines. The genome readmapping pipelines were developed using the nf-core tooling ( Ewels *et al.*, 2020) and use MultiQC ( Ewels *et al.*, 2016), and make extensive use of the Conda package manager, the Bioconda initiative ( Grüning *et al.*, 2018), the Biocontainers infrastructure ( da Veiga Leprevost *et al.*, 2017), and the Docker ( Merkel, 2014) and Singularity ( Kurtzer *et al.*, 2017) containerisation solutions. The genome was also analysed within the BlobToolKit environment ( Challis *et al.*, 2020) and BUSCO scores ( Manni *et al.*, 2021) were calculated.

Table 4 contains a list of relevant software tool versions and sources.

**Table 4.** Software tools: versions and sources.

Software tool	Version	Source
BlobToolKit	4.2.1	https://github.com/blobtoolkit/blobtoolkit
BUSCO	5.3.2	https://gitlab.com/ezlab/busco
bwa-mem2	2.2.1	https://github.com/bwa-mem2/bwa-mem2
Cooler	0.8.11	https://github.com/open2c/cooler
Gfastats	1.3.6	https://github.com/vgl-hub/gfastats
Hifiasm	0.16.1-r375	https://github.com/chhylp123/hifiasm
HiGlass	1.11.6	https://github.com/higlass/higlass
Merqury	MerquryFK	https://github.com/thegenemyers/MERQURY.FK
MitoHiFi	2	https://github.com/marcelauliano/MitoHiFi
OATK	0.1	https://github.com/c-zhou/oatk
PretextView	0.2	https://github.com/wtsi-hpag/PretextView
purge_dups	1.2.5	https://github.com/dfguan/purge_dups
sanger-tol/genomenote	v1.0	https://github.com/sanger-tol/genomenote
sanger-tol/readmapping	1.1.0	https://github.com/sanger-tol/readmapping/tree/1.1.0
YaHS	1.2a.2	https://github.com/c-zhou/yahs

### Wellcome Sanger Institute – Legal and Governance

The materials that have contributed to this genome note have been supplied by a Darwin Tree of Life Partner. The submission of materials by a Darwin Tree of Life Partner is subject to the **‘Darwin Tree of Life Project Sampling Code of Practice’**, which can be found in full on the Darwin Tree of Life website here. By agreeing with and signing up to the Sampling Code of Practice, the Darwin Tree of Life Partner agrees they will meet the legal and ethical requirements and standards set out within this document in respect of all samples acquired for, and supplied to, the Darwin Tree of Life Project.

Further, the Wellcome Sanger Institute employs a process whereby due diligence is carried out proportionate to the nature of the materials themselves, and the circumstances under which they have been/are to be collected and provided for use. The purpose of this is to address and mitigate any potential legal and/or ethical implications of receipt and use of the materials as part of the research project, and to ensure that in doing so we align with best practice wherever possible. The overarching areas of consideration are:

•   Ethical review of provenance and sourcing of the material

•   Legality of collection, transfer and use (national and international)

Each transfer of samples is further undertaken according to a Research Collaboration Agreement or Material Transfer Agreement entered into by the Darwin Tree of Life Partner, Genome Research Limited (operating as the Wellcome Sanger Institute), and in some circumstances other Darwin Tree of Life collaborators.

## Data Availability

European Nucleotide Archive: *Comarum palustre*. Accession number PRJEB61204; https://identifiers.org/ena.embl/PRJEB61204 ( Wellcome Sanger Institute, 2023). The genome sequence is released openly for reuse. The *Comarum palustre* genome sequencing initiative is part of the Darwin Tree of Life (DToL) project. All raw sequence data and the assembly have been deposited in INSDC databases. Raw data and assembly accession identifiers are reported in Table 1. Members of the Royal Botanic Gardens Kew Genome Acquisition Lab are listed here: https://doi.org/10.5281/zenodo.12625079. Members of the Plant Genome Sizing collective are listed here: https://doi.org/10.5281/zenodo.7994306. Members of the Darwin Tree of Life Barcoding collective are listed here: https://doi.org/10.5281/zenodo.12158331 Members of the Wellcome Sanger Institute Tree of Life Management, Samples and Laboratory team are listed here: https://doi.org/10.5281/zenodo.12162482. Members of Wellcome Sanger Institute Scientific Operations: Sequencing Operations are listed here: https://doi.org/10.5281/zenodo.12165051. Members of the Wellcome Sanger Institute Tree of Life Core Informatics team are listed here: https://doi.org/10.5281/zenodo.12160324. Members of the Tree of Life Core Informatics collective are listed here: https://doi.org/10.5281/zenodo.12205391. Members of the Darwin Tree of Life Consortium are listed here: https://doi.org/10.5281/zenodo.4783558.
