# Supplementary material for: The genome sequence of the marsh cinquefoil, Comarum palustre L., also known as Potentilla palustris (L.) Scop. (Rosaceae)
Source: Wellcome Open Res. 2024 Sep 5;9:518. [Version 1] doi: 10.12688/wellcomeopenres.23016.1 (PMC11549540; doi:10.12688/wellcomeopenres.23016.1)
Supplement: Peer review history

# Reviewer report: Reviewer response for version 1

**DOI:** 10.21956/wellcomeopenres.25346.r108801

**Published:** 2024-11-08

**Authors:** Matsapume Detcharoen

**Affiliations:** 1 Prince of Songkla University, Hat Yai, Thailand

Christenhusz and his team sequenced and assembled the genome of *Comarum palustre*. The plant’s taxonomic status is under debate due to complex relationships with the genus *Potentilla*. The team used Hifi and Hi-C sequencing technologies to produce 528.90 Mb genome with high coverage. The final assembly assigned the scaffolds to 21 chromosomal pseudomolecules, supporting the species’ hexaploidy. Other results are fine, including 98% BUSCO completeness and low contamination. This work is valuable for future taxonomic and evolutionary studies of Potentilla and other related genera. The study is well designed and presents its findings in a clear manner.

Are sufficient details of methods and materials provided to allow replication by others?

Yes

Is the rationale for creating the dataset(s) clearly described?

Yes

Are the datasets clearly presented in a useable and accessible format?

Yes

Are the protocols appropriate and is the work technically sound?

Yes

Reviewer Expertise:

molecular ecology and symbiosis

I confirm that I have read this submission and believe that I have an appropriate level of expertise to confirm that it is of an acceptable scientific standard.

# Reviewer report: Reviewer response for version 1

**DOI:** 10.21956/wellcomeopenres.25346.r103920

**Published:** 2024-11-05

**Authors:** Sara L Martin

**Affiliations:** 1 Agriculture and Agri-Food Canada, Ottawa, Canada

This article presents a high quality assembly of the species known as *Comarum palustre* L. or *Potentilla palustris* (L.) Scop depending on the circumscription of the genus *Potentilla* – an issue that requires additional work to resolve. The species is known to exist in multiple cytotypes, however, the authors completed flow cytometry on the sequenced individual and determined that the genome size was 680 Mb. The genome sequencing data used was PacBio HiFi long reads at 35X coverage, Hi-C data with 345 X coverage, and approximately 60 million RNA reads. This data was assembled using Hifiasm, duplicates purged with purge_dups and scaffolding completed with YaHS. This final assembly contained only 86 scaffolds and totaled 528.9 Mb or 78% of the expected size. Curation of this assembly resulted in 21 chromosomal pseudomolecules, as expected for a 2n = 6x = 42 accession of this species. The quality metrics for the assembly are good with, for example, a 95% BUSCO completion score. The authors also assembled the chloroplast and the mitochondria. The article is well written and clear with good figures and tables that help the reader to understand the quality of the genome. The methods and references are appropriate.

 I have three minor comments. I would have liked to see a different (smaller) standard used in the flow cytometry as the size difference between the 2.2GB standard and the 680Mb sample could lead to some degree of inaccuracy in the estimate. However, the difference is just over the three times rule so it is marginal and not unacceptable. I would have liked for the authors to compare this size to other known diploid, tetraploid and hexaploid relatives for context since the cytotype of the individual seems to have been an open question. However I understand the primary focus of this work is presenting the genome rather than any comparative work. Finally, I would have liked to see that the authors had performed a chromosome count on the plant sequenced in order to confirm that the individual was 2n = 6x = 42 and was indeed expected to result in an assembly with 21 chromosomes. Overall, these issues are minor and the work is of an excellent quality. I am particularly happy to see that a herbarium record was created so that it will be available to back up the identification of the species, that the barcode information and raw data are already available, and with the level of detail given in the methods such as the versions of and links to all software used.

 This genome will be an excellent resource for furthering our understanding of the related crop *Fragaria* and for understanding polyploidization within this group of species. I can see several interesting papers that would be possible using this genome for comparative genomics.

Are sufficient details of methods and materials provided to allow replication by others?

Yes

Is the rationale for creating the dataset(s) clearly described?

Yes

Are the datasets clearly presented in a useable and accessible format?

Yes

Are the protocols appropriate and is the work technically sound?

Yes

Reviewer Expertise:

My main interest is in genome evolution including polyploidization. I have expertise in genome assembly and analysis, population genomics, hybridization, and the evolution of herbicide resistance.

I confirm that I have read this submission and believe that I have an appropriate level of expertise to confirm that it is of an acceptable scientific standard.
